# Targeting Eukaryotic mRNA Translation by *Legionella pneumophila*

**DOI:** 10.3389/fmolb.2020.00080

**Published:** 2020-04-29

**Authors:** Yury Belyi

**Affiliations:** Gamaleya Research Centre for Epidemiology and Microbiology, Moscow, Russia

**Keywords:** *Legionella*, protein synthesis, inhibition, elongation factor eEF1A, glycosylation

## Abstract

*Legionella* is a gram-negative microorganism and an infectious agent of pneumonia in humans. It is an intracellular pathogen and multiplies in different eukaryotic cells like amoebae, ciliated protozoa, macrophages, monocytes, and lung epithelial cells. Proliferation of *L. pneumophila* in eukaryotic cells depends on its type 4 secretion system, which delivers an arsenal of bacterial effector proteins to cytoplasm of its host. Once within the cytoplasm, effectors modify a broad range of host activities, including mRNA translation. Translation is inhibited by *Legionella* through the action of several effector proteins including Lgt1, Lgt2, Lgt3, SidI, LegK4, SidL, and RavX. Lgt1-3 and SidI target elongation factors: Lgt1-3 mono-glucosylate elongation factor eEF1A, while SidI binds eEF1A, and eEF1Bγ. Effector LegK4 inhibits protein synthesis by phosphorylating Hsp70 proteins, while SidL and RavX have no defined targets in protein synthesis machinery thus far. In addition to direct inhibition of protein synthesis, SidI also affects the stress response, whereas Lgt1-3 – unfolded protein response and cell-cycle progression of host cells. Whether manipulation of these processes is linked to canonical or non-canonical function(s) of targeted elongation factors remains unknown.

## Introduction

Protein synthesis is vitally important to eukaryotic cells. Therefore, it is not surprising that mRNA translation has been targeted by pathogens throughout evolution. The first bacterial molecule capable of inhibiting protein synthesis, identified in the late 1950s and early 1960s, was the “diphtheriae toxin” of *Corynebacterium diphtheriae* (Strauss and Hendee, 1959; Kato and Pappenheimer, 1960). Some years later, another toxin with similar activity was found in cultures of *Pseudomonas aeruginosa* and was named “exotoxin A” (Liu, 1966). Soon thereafter, the molecular mechanism of translational inhibition was revealed for both identified toxins, which involved the mono-adenosine 5′-diphosphate (ADP) ribosylation of eukaryotic translational elongation factor 2 (eEF2; Honjo et al., 1968; Gill et al., 1969; Iglewski and Kabat, 1975). The site of modification was determined to be a diphthamide residue, which involved post-translationally modified histidine-699 of eEF2 (Van Ness et al., 1980).

In subsequent studies, multiple research groups were able to show that proliferation of certain medically important microorganisms like *Shigella*, *Salmonella*, *Chlamydia* or *Legionella* within host cells resulted in the inhibition of eukaryotic protein synthesis (Hale and Formal, 1980, 1981; McCusker et al., 1991; Ohmer et al., 2019). In *Shigella* and *Salmonella*, observed cytotoxic effects were attributed to the production of cytotoxins, Shiga or Shiga-like toxins by the pathogens (Brown et al., 1980; Koo et al., 1984). The latter toxins specifically cleaved 28S rRNA to prevent the elongation of peptide chains on eukaryotic ribosomes (Endo et al., 1988). However, observed translational inhibition in target cells by *Legionella* was not explained until our knowledge of bacterial secretion systems improved (Segal et al., 1998; Vogel et al., 1998). It turned out that *per se* non-toxic protein effectors, been delivered directly into eukaryotic cytoplasm by specialized secretion machinery, became powerful virulence factors with diverse intracellular activities. These advancements revolutionized the field of bacterium-host interactions and created a platform for the identification of novel toxic proteins and the elucidation of sophisticated virulence mechanisms.

## Intracellular Biology of *L. pneumophila*

*Legionella pneumophila* is a gram-negative bacterium and an infectious agent of legionellosis, a most known form of which (Legionnaires’ Disease) is characterized by severe pneumonia in humans (McDade et al., 1977). In natural environment the pathogen multiplies in a free-living unicellular organism like amoebae and ciliated protozoa (Rowbotham, 1983). During the infection process in humans the microorganisms predominantly invade macrophages, monocytes and lung epithelial cells (Richards et al., 2013). After uptake by host cells, legionellae multiply within a specialized phagosome-derived replicative vacuole, which avoids fusion with the lysosome and subsequent degradation (Isberg et al., 2009). Formation of a replicative vacuole by *L. pneumophila* is dependent upon the bacterial type 4 secretion system (T4SS), which translocates a plethora of bacterial effector proteins to the eukaryotic target cell. The highly specialized activities of this arsenal of *Legionella* factors are prerequisites for the successful proliferation of the infectious agent within its host (Escoll et al., 2016).

The range of eukaryotic organelles and host processes targeted by the *Legionella* effectors is amazingly broad (Escoll et al., 2016). The largest group of *Legionella* effectors manipulates eukaryotic small GTPases, which are involved in vesicular trafficking and membrane maturation in host cells [reviewed in Goody and Itzen (2013), Sherwood and Roy (2013), Isaac and Isberg (2014), Hilbi et al. (2017), Spano and Galan (2018)]. However, in addition to modifying endocytic machinery, a vast number of other cellular processes are affected throughout *Legionella* replication within host cells. These include apoptosis (Misch, 2016), autophagy (Sherwood and Roy, 2016; Siqueira et al., 2018), DNA transcription (Li et al., 2013; Rolando et al., 2013; Lee and Machner, 2018; Schuelein et al., 2018; Von Dwingelo et al., 2019), cytoskeleton functioning (Guo et al., 2014; Michard et al., 2015; Roy et al., 2017; He et al., 2019), mitochondrial dynamics (Arasaki et al., 2017), and phospholipid biosynthesis (Viner et al., 2012). Importantly, several *L. pneumophila* effectors have been shown to inhibit eukaryotic protein synthesis by targeting mRNA translation either by directly attacking translational factors or by phosphorylation of ribosome-associated chaperones (Belyi et al., 2006; Shen et al., 2009; Moss et al., 2019).

## *L. pneumophila* Effectors That Target Protein Synthesis in Eukaryotic Cell

The first identified *L. pneumophila* effector that has been shown to inhibit protein synthesis was glucosyltransferase Lgt1 [*Legionella* glucosyltransferase 1, (GeneBank identification code is Lpg1236)] (Belyi et al., 2003). Later, *in silico* analysis of available genomic sequences from *L. pneumophila* strains revealed that several open reading frames exhibited significant sequence similarity to Lgt1. Based on their amino acid sequences, gene products were grouped into three subfamilies [Lgt1, Lgt2 (Lpg2862), and Lgt3 (Lpg1488)]. Some strains of *L. pneumophila* (e.g., Philadelphia-1) contained *lgt1*, *lgt2*, and *lgt3*, whereas others (e.g., Paris, Corby, Lens) possessed only *lgt1* and *lgt3* (Belyi et al., 2008). Interestingly, the prevalence of Lgt2 was higher within clinical strains of the bacteria than in environmental *L. pneumophila* isolates (Sadretdinova et al., 2012). Therefore, one can speculate that the addition of Lgt2 to the repertoire of glucosyltransferases in *Legionella* increases the virulence of the pathogen and/or broadens its potential host range.

Production of Lgt1 and Lgt2 by microbial cells was strongly increased during the stationary phase of bacterial growth *in vitro*, while Lgt3 was detectable in throughout the pre-logarithmic growth phase. Similar results were obtained *in vivo*, when the protozoan *Acanthamoeba castellanii* was used as a host of *L. pneumophila*. In this study, levels of mRNA encoding Lgt1 were maximal at late timepoints of bacteria-amoeba co-infection, while *lgt3* was expressed mainly at the initial stage of the interaction between *Legionella* and *A. castellanii* (Belyi et al., 2008). These experiments suggested that glucosyltransferase activity was differentially regulated in *L. pneumophila* and indicated that each enzyme had specific role in promoting bacterial virulence. Speculatively, Lgt3 could be important for the initiation of the infection cycle, while Lgt1-2 might be necessary for *Legionella* egress from the host cell.

The crystal structure of Lgt1 is available (Hurtado-Guerrero et al., 2010; Lü et al., 2010). The effector is classified as a GT-A type glucosyltransferase family protein in the carbohydrate-active enzymes database http://www.cazy.org/GT88.html (Lombard et al., 2014). Several conserved amino acid residues of the catalytic core have been identified, including two aspartic amino acid residues (D-246 and D-248), which form a DXD motif that is typical of glycosyltransferases. The motif is crucial for divalent cation binding and coordination of the co-substrate (Busch et al., 1998; Wiggins and Munro, 1998). Lgt1 also possesses a flexible loop at its COOH-terminus, which is important for the proper arrangement of the donor substrate binding site, the accommodation of uridine diphospho (UDP)-glucose in the catalytic center and the release of reaction products after catalysis. Lgt1 uses UDP-glucose as a donor for the glucosylation reaction. The first identified target of Lgt1-3 was reported to be eukaryotic elongation factor eEF1A.

**FIGURE 1:**
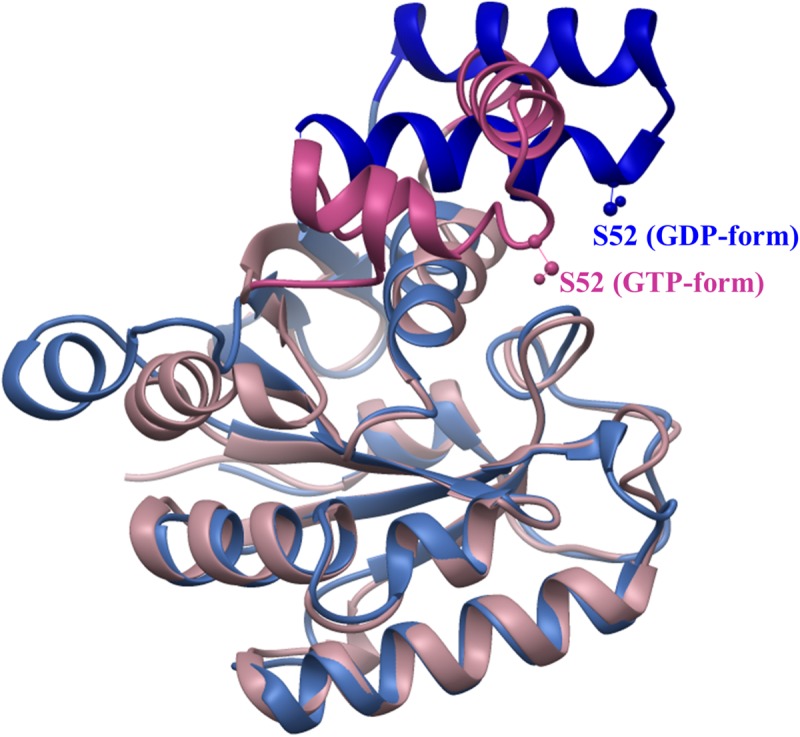
Conformational change of archaeal elongation factor 1A in GDP- and GTP-bound states. Structures of G-domains of aEF1A from *Sulfolobus solfataricus* in complex with GDP (1SKQ) and *Aeropyrum pernix* in complex with GTP (3AGJ) were aligned using USCF Chimera (Pettersen et al., 2004). Serine-52 (analog of serine-53 in eEF1A, which is glucosylated by Lgt1-3) is shown in “ball and stick” representation. Regions of aEF1As subjected to major change are drawn in dark blue (GDP-bound) and orchid (GTP-bound) colors, while regions of similar structure are colored in sky-blue (GDP-bound) and pink (GTP-bound).

When mRNA is translated, eEF1A cycles between forming a translationally active ternary complex (eEF1A•GTP•aminoacyl-tRNA) and its inactive, GDP-bound conformation. The NH_2_-terminal G-domain of eEF1a, which is necessary for GTP binding and hydrolysis, plays a major role in the transition between inactive and active forms. Lgt1-3 modify an amino acid residue serine-53 located near the Switch-1 region of the G domain of the elongation factor that lies within a protruding loop that connects helices A^∗^ and A’ (Pedersen et al., 2001). Our studies have shown that eEF1A within a ternary complex, or truncated versions of the elongation factor were glucosylated several orders of magnitude higher than the full-length, apo-form of eEF1A (Belyi et al., 2009; Tzivelekidis et al., 2011). These data indicate that glucosylation efficiency appears to depend on the specific conformation of the factor. Indeed, the Switch-1 region undergoes conformational change as it alternates between GDP- and GTP-bound states (Figure 1). This change in conformation may influence modification rates and confer “super-specificity” to Lgt1-3-induced glucosylation, since the *Legionella* effectors are then able to discriminate and glucosylate not every, but preferentially translationally competent form of eEF1A within host cells.

Another substrate of Lgt1-3 is Hbs1 [Hsp70 subfamily B suppressor 1 (Nelson et al., 1992)]. Hbs1 is a conserved protein that can be found in diverse eukaryotic organisms ranging from yeast to humans and is implicated in the recycling of ribosomes stalled on an aberrant mRNA (Hilal et al., 2016). Yeast Hbs1 was modified on serine-213, located within a region structurally similar to the substrate sequence in eEF1A (Belyi et al., 2009).

Eukaryotic substrates modified by Lgt1-3 (eEF1A and Hbs1) include crucial components of the translational machinery. Not surprisingly, addition of this effector protein to *in vitro* reticulocyte translational extracts resulted in the dose-dependent inhibition of protein synthesis. Furthermore, delivery of the protein into mammalian cells, or expression of the corresponding genes in *Saccharomyces cerevisiae*, resulted in eEF1A glucosylation, the inhibition of protein synthesis, and cell death. Yeast strain containing eEF1A S53A but not Hbs1 S213A was insensitive to glucosylation and did not die in the presence of intracellular Lgt1 (Belyi et al., 2012). The latter experiment indicates that elongation factor eEF1A is the major target of the *Legionella* Lgt1 enzyme that causes toxicity in yeast.

SidI (Lpg2504) is another *Legionella* effector that has been shown to inhibit protein synthesis and kill eukaryotic cells (Shen et al., 2009; Joseph et al., 2020). This protein was identified during a screen of *L. pneumophila* genes capable of producing toxic effects in *S. cerevisiae*. Subsequently, the authors were able to demonstrate that SidI directly binds to both eEF1A and eEF1Bγ. The binding efficiency of wild type and point-mutated SidI correlated neither with its toxicity to eukaryotic cells nor with its inhibitory effect on mRNA translation *in vitro* (Shen et al., 2009). Moreover, the COOH-terminal fragment of the effector, shown to be able to bind eEF1A, only modestly inhibited *in vitro* translation compared to the full-sized molecule (Joseph et al., 2020). These data suggested that in addition to binding, the effector might possess another type of biochemical activity (e.g., enzymatic) that is required for its observed biological effects.

Indeed, using structure-predicting software, researchers have been able to demonstrate that SidI and GT-B fold glycosyltransferases are structurally similar. In accordance with this observation, the SidI protein has been shown to have the capacity to cleave GDP-mannose and, to a much lesser extent, UDP-glucose (Joseph et al., 2020). However, whether SidI can glycosylate any targets is not clear, since treatment of eEF1A with the effector did not result in any type of posttranslational modification of the protein (Shen et al., 2009). Interestingly, protein synthesis inhibition, accomplished by SidI is potently regulated by a metaeffector, *L. pneumophila* protein termed MesI (Lpg2505), which can bind to and significantly suppress enzymatic activity of the effector (Joseph et al., 2020).

LegK4 (lpl0262) is a protein kinase effector produced by *Legionella* that is capable of inhibiting protein synthesis in eukaryotic cells (Barry et al., 2013; Flayhan et al., 2015). LegK4 has been experimentally shown to be capable of phosphorylating conserved threonine residue of members of Hsp70 chaperone family both *in vitro* and *in vivo*. This residue (T-492 in Ssa1 of *S. cerevisiae*) is located within substrate binding domain and its modification by the protein kinase results in reduced chaperone ATPase activity and the concomitant inhibition of the refolding ability. Phosphorylation and subsequent inactivation of Hsp70 enhances its association with ribosomes and reduces global levels of translation. These data suggest a mechanism in which Hsp70 molecules, after being phosphorylated by LegK4, fail to fold nascent polypeptides correctly. Therefore, they remain associated with polysomes longer than usual, and block protein synthesis (Moss et al., 2019).

The least-studied effectors capable of inhibiting eukaryotic translation *in vivo* are SidL (Lpg0437; Fontana et al., 2011) and RavX (Lpg1489; Barry et al., 2013). Both proteins were shown to have the capacity to kill eukaryotic cells. However, the targets within eukaryotic cells and mechanisms of the translational inhibition of these effectors have not been elucidated. Surprisingly, the toxicity of SidL to yeast was completely alleviated by overexpression of profilin, one of the major cytoskeleton-related proteins. In other experiments direct interaction of the effector with actin and subsequent inhibition of actin polymerization were demonstrated (Guo et al., 2014). Bearing in mind the fact that actin cytoskeleton and components of protein synthesis machinery are functionally linked (Gross and Kinzy, 2005; Pittman et al., 2009), it would be interesting to explore the mechanism of inhibition of mRNA translation by SidL in relation to its effect upon eukaryotic cytoskeleton.

## Cell Killing by *Legionella* Effectors and Beyond

**FIGURE 2:**
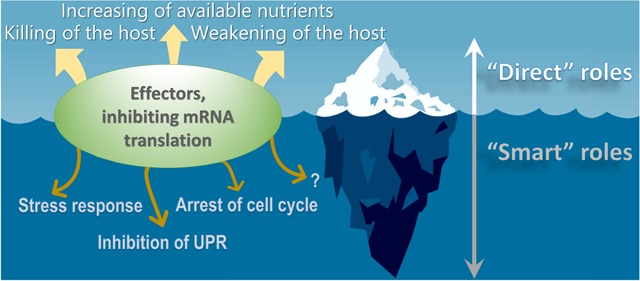
Different roles of *Legionella* effectors inhibiting eukaryotic mRNA translation. The bacterial inhibitors of eukaryotic protein synthesis may facilitate decrease in the translation of eukaryotic transcripts thus providing a large pool of unused nutrients to *Legionella*, weakening of antibacterial activity and ultimately – killing of the host. The above examples represent “direct” roles of the effectors. However, the latter proteins might also play “smart” roles in the virulence of *Legionella* that are aimed at delicately manipulating host cell functioning for the benefit of the pathogen, including regulation of stress response, suppressing of unfolded protein response, arrest of cell cycle progression and possibly some other processes, which are unknown to date.

The significance of effector-induced protein synthesis inhibition for the pathogenesis of *Legionella* infection remains unclear. It has been speculated that the action of bacterial inhibitors of eukaryotic mRNA translation strongly decreases cellular metabolism, and correspondingly, antibacterial activity. This makes weakened host cells more susceptible to invading bacteria (Figure 2). Another possible explanation states that global decreases in the translation of eukaryotic transcripts may provide a large pool of unused amino acids and other nutrients to *Legionella*. Finally, at late stages of the intracellular life cycle, *Legionella* escape from its host requires the killing of the eukaryotic cell. Translation-targeting effectors may facilitate such a killing of the host. The above possibilities can be attributed to as “direct” roles of the effectors (Belyi et al., 2013).

Several lines of evidence suggest, however, that the biological importance of effectors should not be oversimplified. Accumulated data show that virulence factors might have “smart” roles that are aimed at delicately manipulating of host cell functioning for the benefit of the pathogen (Figure 2). In this case, observed cytotoxicity may be a side effect of some other pro-bacterial consequences of translational arrest (Ensminger and Isberg, 2009). One example of a smart role of *Legionella* effectors was reported in the investigation of the regulation of the stress response by SidI.

Stress shock response is a fundamental mechanism necessary for eukaryotic cell survival within a variety of harmful environments. The stress response in mammalian cells is controlled by a multi-component complex that consists of heat shock transcription factor 1 (Hsf1), eEF1A and non-coding RNA molecules (heat shock RNA 1, Hsr1; Shamovsky et al., 2006). Hsf1 is able to bind specific promoters (heat shock elements, Hse) and thus, induces the production of a panel of heat shock proteins necessary for rescuing eukaryotic cells that are experiencing unfavorable conditions (Sarge et al., 1991). Infection of macrophage-like cells with virulent *L. pneumophila* or transfection of eukaryotic cells with SidI-coding plasmids resulted in an elevated eukaryotic stress response, which researchers detected by observing elevated levels of the Hfs1/eEF1A complex, increased binding of Hsf1 to Hse and the stimulation of *hsp70* expression (Shen et al., 2009). These results indicate that Hsf1 is activated during *L. pneumophila* infection and SidI, which was initially shown to suppress protein synthesis, mediates its activation.

Another process that is influenced by *Legionella* infection is the unfolded protein response (UPR). The UPR is initiated by the eukaryotic cell to cope with the accumulation of misfolded proteins within the lumen of the endoplasmic reticulum (ER; Hetz and Papa, 2018). A branch of the UPR is also linked to the innate immune response (Hasnain et al., 2012). The consequences of activating the UPR include the inhibition of global protein synthesis, upregulation of the production of ER stress proteins (e.g., luminal chaperone BiP), and the initiation of apoptotic (e.g., through induction of CHOP) and proinflammatory (e.g., activation of NF-kB) programs.

*Legionella pneumophila* was shown to inhibit the UPR via multiple mechanisms (Treacy-Abarca and Mukherjee, 2015). To elucidate means by which UPR was manipulated by *L. pneumophila*, experiments investigating IRE1, a known sensor of the UPR were conducted. Under conditions of ER stress, activated IRE1 removes an intron from XBP1 mRNA, an UPR intermediate. Thus, a spliced variant is formed that acts as a transcription factor and enhances transcription of the ER chaperone gene. The intracellularly-multiplying, wild-type *L. pneumophila* strain efficiently blocked effects of the production of the splice variant. The genetic inactivation of T4SS of *Legionella* or the five translational effectors (i.e., Δ*5*: Lgt1-3, SidI, and SidL) restored splicing activity in host cells, which was able to be blocked again by complementing the Δ*5* mutant with plasmids encoding Lgt2 or Lgt3 (Hempstead and Isberg, 2015; Treacy-Abarca and Mukherjee, 2015).

*Legionella* multiplication is dependent upon the life cycle of its host. In particular, it has been shown that host cells in G1 and G2/M phases facilitate bacterial replication, while eukaryotic cells in S phase provide an environment that hinders *L. pneumophila* replication (de Jesús-Díaz et al., 2017). The wild type, but not the Δ*5* strain, was able to arrest host cell-cycle progression in macrophages and amoebae. Strikingly, failure of the Δ*5* strain to control cell-cycle progression was able to be reversed by transfecting the host with Lgt3-encoding plasmid. Moreover, cell cycle arrest was initiated by the ectopic expression of a single glucosyltransferase protein, either Lgt1 or Lgt3 (Sol et al., 2019).

## Discussion

Modification of the activities of key translation factors appears to be an important virulence mechanism that aids proliferation of *Legionella* within its eukaryotic host. Given that translation factors have both canonical and non-canonical functions, their regulation allows the pathogen to directly or indirectly influence an array of processes within the host cell. This should be kept in mind while studying intracellular biology of *L. pneumophila* and pathogenesis of the infectious disease. Although this type of knowledge mainly is in the realm of basic science, it will certainly facilitate the development of new therapeutics, vaccines and diagnostic methods.

The high degree of specificity and activity of bacterial virulence factors make them powerful probes that facilitate the dissection of the functions of their eukaryotic targets. There are numerous examples of how the use of virulence factors have enhanced our knowledge of host biology. In particular, the study of Rho protein biochemistry, G-protein-guided signal transduction and retrograde transport have been elucidated by the study of glucosylating *Clostridium difficile* toxins, *Pasteurella multocida* toxin and Shiga toxin, respectively. Therefore, the addition of *Legionella* effectors to this experimental toolbox may facilitate the discovery of novel functions of different eukaryotic proteins and may shed light on their pathways. Elongation factors are emerging as types of eukaryotic proteins whose functions have become more obvious as a result of the study of proteins produced by *L. pneumophila*.

## Conflict of Interest

The authors declare that the research was conducted in the absence of any commercial or financial relationships that could be construed as a potential conflict of interest.
